# Development of plant-produced protein body vaccine candidates for bluetongue virus

**DOI:** 10.1186/s12896-017-0370-5

**Published:** 2017-05-30

**Authors:** Albertha R. van Zyl, Ann E. Meyers, Edward P. Rybicki

**Affiliations:** 1 Department of Molecular and Cell Biology, University of Cape Town, Private Bag X3, Rondebosch, 7700 South Africa; 2 Institute of Infectious Disease and Molecular Medicine, Faculty of Health Sciences, University of Cape Town, Observatory, Cape Town, 7925 South Africa

**Keywords:** Bluetongue virus, Protein body, *Nicotiana benthamiana*, Vaccine, Zera®

## Abstract

**Background:**

Bluetongue is a disease of domestic and wild ruminants caused by bluetongue virus serotypes (BTV), which have caused serious outbreaks worldwide. Commercially available vaccines are live-attenuated or inactivated virus strains: these are effective, but there is the risk of reversion to virulence or reassortment with circulating strains for live virus, and residual live virus for the inactivated vaccines. The live-attenuated virus vaccines are not able to distinguish naturally infected animals from vaccinated animals (DIVA compliant). Recombinant vaccines are preferable to minimize the risks associated with these vaccines, and would also enable the development of candidate vaccines that are DIVA-compliant.

**Results:**

In this study, two novel protein body (PB) plant-produced vaccines were developed, Zera®-VP2ep and Zera®-VP2. Zera®-VP2ep contained B-cell epitope sequences of multiple BTV serotypes and Zera®-VP2 contained the full-length BTV-8 VP2 codon-optimised sequence. In addition to fulfilling the DIVA requirement, Zera®-VP2ep was aimed at being multivalent with the ability to stimulate an immune response to several BTV serotypes. Both these candidate vaccines were successfully made in *N. benthamiana* via transient *Agrobacterium*-mediated expression, and in situ TEM analysis showed that the expressed proteins accumulated within the cytoplasm of plant cells in dense membrane-defined PBs. The peptide sequences included in Zera®-VP2ep contained epitopes that bound antibodies produced against native VP2. Preliminary murine immunogenicity studies showed that the PB vaccine candidates elicited anti-VP2 immune responses in mice without the use of adjuvant.

**Conclusions:**

These proof of concept results demonstrate that Zera®-VP2ep and Zera®-VP2 have potential as BTV vaccines and their development should be further investigated.

**Electronic supplementary material:**

The online version of this article (doi:10.1186/s12896-017-0370-5) contains supplementary material, which is available to authorized users.

## Background

Bluetongue (BT) is a non-contagious, arthropod-borne viral disease that affects ruminants [1]. BT was first recognised and described in South Africa after the introduction of fine-wool sheep from Europe over 200 years ago [2, 3]. The classical form of BT is seen in sheep; however, cattle are also a natural reservoir for the virus [1]. The causative agent of BT is bluetongue virus (BTV), the type species of the genus *Orbivirus* in the family *Reoviridae* [4]. BTV is mostly transmitted by adult females of the haematophagous midges that belong to the genus *Culicoides* [5].

Since 1998 BTV has become one of the most widespread animal pathogens, as it has spread to areas that were previously free of the virus [6]. Outbreaks of BT occur when susceptible sheep are introduced into BTV-endemic regions or when the virus spreads to naïve sheep populations at the interface of endemic and non-endemic regions [7]. In 2006 BTV serotype 8 (BTV-8) was detected in northern Europe (Netherlands, Belgium, Germany and the north of France); this was the first time that BTV had been detected beyond the latitude of 52 °N. In subsequent outbreaks the northernmost limits of BTV moved beyond 54 °N [2, 8].

A number of vaccines have been developed against BT. These include inactivated whole virus vaccines, live attenuated virus vaccines (modified live virus vaccines), recombinant vaccines and virus-like particle (VLP) vaccines. While these vaccines have various advantages and disadvantages, only attenuated virus vaccines and some inactivated vaccines are presently commercially available [9–12]. A vaccine produced by Onderstepoort Biological Products (OBP, Pretoria, South Africa) that consists of a mixture of attenuated field strains is widely used in South Africa [13, 14]. However, several side effects including the development of mild clinical symptoms [15], decreased milk production [9] and transplacental infection [16, 17] have been documented with the use of these vaccines. These factors have led to the development of recombinant BTV vaccines. VLP vaccine candidates have been produced using insect cells and more recently, also plant-based expression systems, and were shown to be safe and effective, with vaccinated sheep protected against virus challenge [18–20]. A disadvantage of these however, is that they are protective against only one of the 27 BTV serotypes, unless they are administered as a combination of VLPs produced against different serotypes, thereby increasing cost of the vaccines. Furthermore, with the use of VLP vaccines, one is not able to distinguish infected from vaccinated animals (DIVA) when using current commercially available diagnostic techniques which rely on the detection of the group specific antigen VP7, [2, 21, 22]. In South Africa 22 of the 27 known BTV serotypes have been detected in the country and it has been found that multiple BTV serotypes co-circulate with each vector season [13]: this demonstrates the necessity for use of a multivalent vaccine for BTV in this region.

The BTV structural protein VP2 is the major serotype-specific antigen of BTV [14, 23]. It has been shown that ≥ 50 μg doses of VP2 obtained from both isolated and purified BTV as well as recombinantly-produced VP2 induced neutralising antibodies protected some, but not all of the sheep that were vaccinated, against viral challenge [14, 24]. Even though these subunit vaccines have been shown to be safe for use in sheep [14], it is desirable to enhance the breadth of immunogenicity of these vaccine candidates.

Epitopes are localised regions on the surfaces of antigens that are involved in recognition by antibodies. These regions also have the ability to elicit an immune response and represent the smallest subunits that can be used therapeutically [25, 26]. Many advantages such as safety, ease of production and analytical control are associated with the use of epitope-based vaccines: with the presentation of specific epitopes a precise immune response can be directed at conserved and highly immunogenic regions of antigens of interest [25]. B-cell epitopes are parts of antigens that are recognised by the variable regions of antibodies [27]. Several epitope-based vaccines have been developed for the treatment of various cancers and the prevention of infectious diseases. Epitope-based vaccines for the treatment of ovarian carcinoma, end-stage cervical cancer and melanoma have been successful and have entered or completed phase I and II clinical trials [28–30]. Furthermore, an epitope-based vaccine derived from the Epstein-Barr virus latency-related antigens has been shown to be immunogenic in pre-clinical trials in mice [31].

The immunogenicity of proteins can be increased by fusion to other immunogenic proteins, such as the hepatitis B core protein [32, 33], by adding adjuvant to the vaccine formulation [34], or by fusion to signal sequences that drive assembly and sequestration of the protein into protein bodies (PBs) [35]. Particulate proteins with repeating sequence motifs, such as PBs, are favoured for uptake by antigen presenting cells, thereby enhancing the immune response [36].

PBs are endoplasmic reticulum (ER)-derived organelles found in maize seeds. These organelles stably store massive amounts of zeins as a source of protein within the ER [37]. Once expressed and targeted to the ER for post translational modification, these zein polypeptides oligomerise in large complexes and eventually self-associate into PBs [38–40]. The proline-rich N-terminal (including a tandem-repeat domain) of one of these zeins - γ-zein - was shown to be important for ER retention and the formation of PBs in both maize seeds and a wide range of eukaryotic cells [41].

Zera® (ZIP Solutions, Spain) is a synthetic peptide generated from the N-terminal proline-rich domain of γ-zein [41]. The Zera® sequence consists of 112 amino acids that include the γ-zein signal peptide and the first 93 amino acids of γ-zein. The complete Zera® sequence contains four regions: a γ-zein ER-targeting signal peptide, 11 hydrophobic non-proline amino acids that contain a CGC motif that is important for packing of protein bodies due to the formation of inter-and intra-chain disulphide bonds, the proline-rich repeat domain containing eight repeats of the hexapeptide PPPVHL which is important for the assembly of PBs and finally, a proline-X (Pro-X region) sequence where proline residues alternate with other amino acids [41, 42]. The main driving forces behind the self-assembly of PBs are hydrophobic interactions between the (PPPVHL)_8_ repeat regions of two or more Zera® chains and inter-chain disulphide bond formation that stabilises and strengthens the oligomers [42].

Zera® fused to several different proteins - including red fluorescent protein [43], enhanced cyan fluorescent protein [44], human growth hormone [44], calcitonin [41] and epidermal growth factor [45] - has been shown to result in the formation of PBs in the leaves of both transiently and stably transformed tobacco plants. It has been shown that fusion of a HPV-16 shuffled E7 protein (E7sh) to Zera® enhanced immunogenicity of the protein; moreover, free protein bodies mixed with free E7sh protein also resulted in an enhanced immune response [36, 46]. More recently, a study by Hofbauer et al. [47] showed that encapsulation of hemagglutinin in PBs resulted in stronger immune responses in mice when compared to responses obtained with soluble antigen.

The antigenic characteristics of BTV VP2 make it an excellent candidate for a BT vaccine. By presenting VP2 to the immune system in particulate form, immunogenicity of the protein should be enhanced, and less of the antigen may be needed for vaccination. Here we report on the development of two novel VP2-based BTV vaccine candidates. For the first vaccine candidate, the putative antibody-binding epitopes of multiple serotypes of VP2 were predicted with *in silico* methods and a synthetic peptide based on the predicted epitopes was constructed. The second vaccine candidate consisted of the full-length plant codon-optimised BTV-8 VP2. Nucleotide sequences representing both the synthetic epitope sequence and the full-length plant codon-optimised BTV-8 VP2 were fused to the Zera®-encoding sequence to drive PB formation when expressed in *N. benthamiana*. In situ TEM analysis of infiltrated leaves was carried out to determine if the Zera®-fused proteins accumulated into PBs. Finally, a preliminary investigation was carried out to assess the immunogenicity of these fusion proteins in mice.

## Results

### Prediction of a putative BTV VP2 epitope

**Fig. 1:**
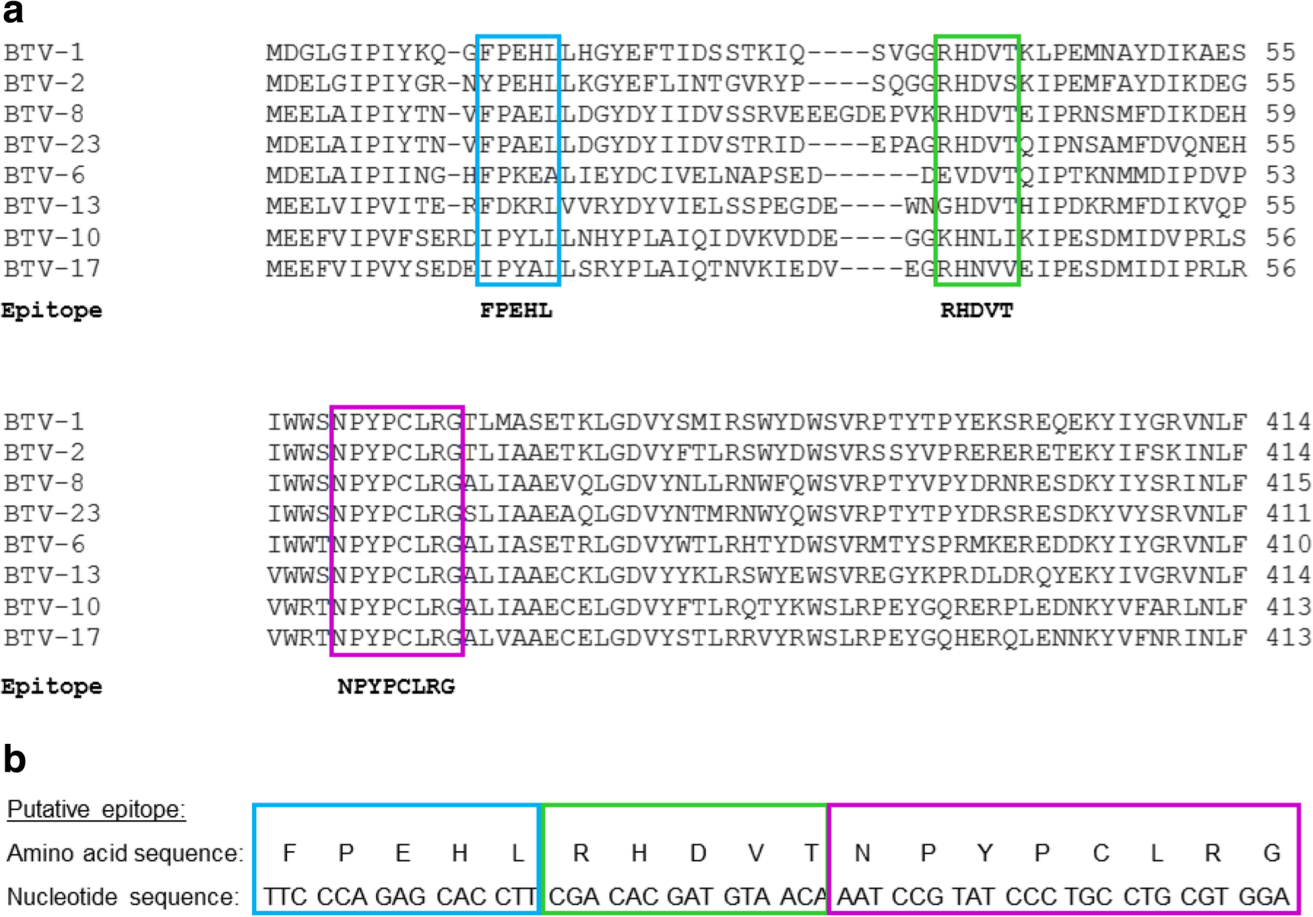
Construction of VP2ep. **a** Selected regions of the multiple alignment of 8 selected BTV VP2 serotype amino acid sequences are shown. The predicted epitope regions corresponding to most of the serotypes are shown below the alignment in *bold and boxed in blue and green*. The homologous region is *boxed in purple*. **b** The putative amino acid and nucleotide sequences of VP2ep

*In silico* prediction of continuous B-cell epitopes was carried out on eight full-length VP2 amino acid sequences that were available on GenBank at the time of the study (http://www.ncbi.nlm.nih.gov/genbank) with COBEpro [27] prediction software available from the Scratch protein server (http://scratch.proteomics.ics.uci.edu/). The 8 VP2 amino acid sequences were aligned and two predicted B-cell epitope regions that were similar for most of the serotypes were selected for the epitope-based vaccine sequence, these epitope regions were not similar to the neutralisation regions (R1 and R2) proposed by deMaula et al. [48]. Two predicted epitope regions (Fig. 1a, blue and green boxes) that showed consensus among most of the amino acid sequences were selected for the design of the putative epitope-based vaccine and a homologous region across all the serotypes was also included in the sequence (Fig. 1a, purple box). In total the putative predicted epitope-based oligomer (Zera®-VP2ep) consisted of 54 bp that translated into 18 amino acids (Fig. 1b). No linkers were included between the putative epitope regions to facilitate folding as these were linear B-cell epitopes that would be folded into Zera® PBs.

### Zera®-VP2ep and Zera®-VP2 expression in *N. benthamiana*

**Fig. 2:**
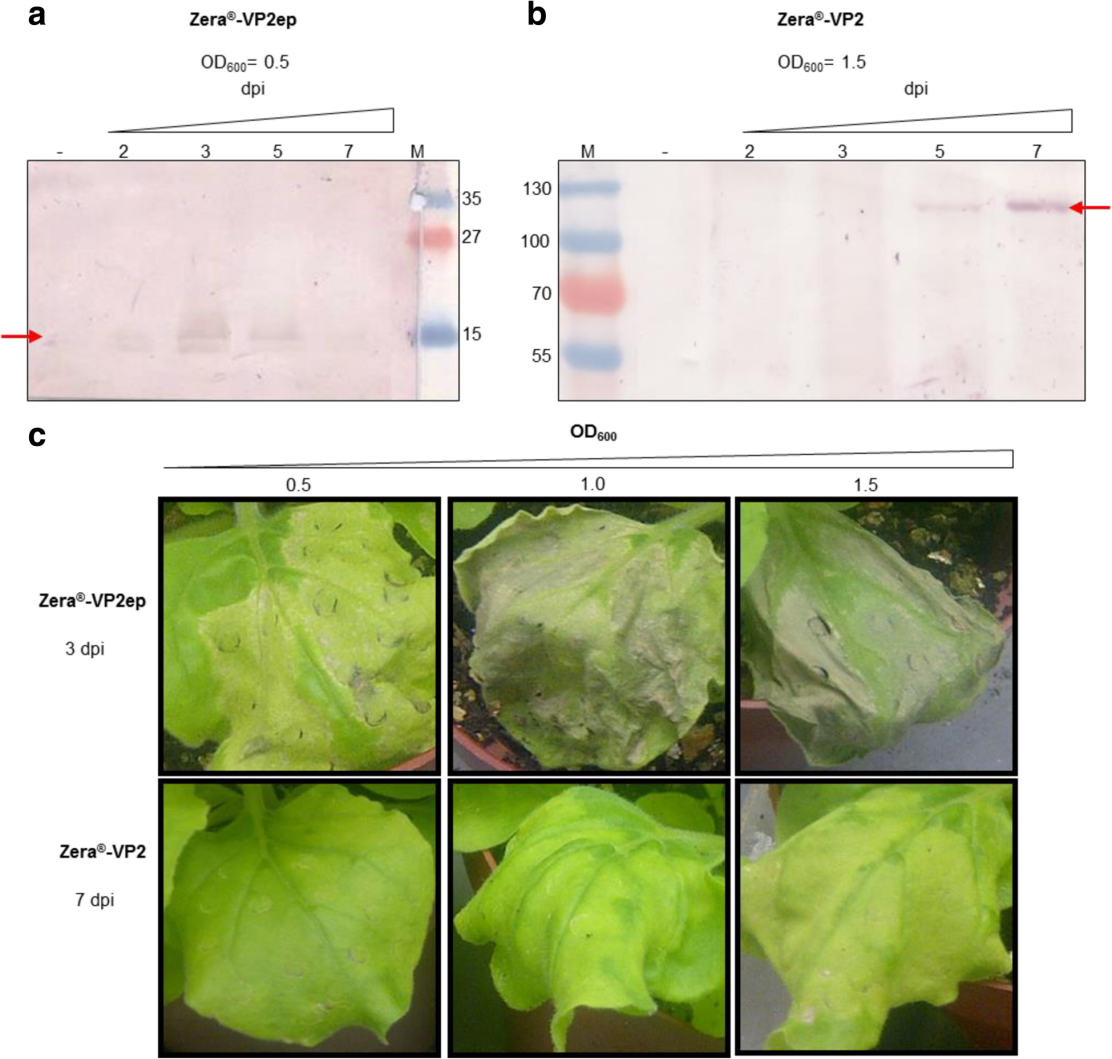
Expression of Zera®-VP2ep and Zera®-VP2 in *N. benthamiana*. Western blots of crude leaf extracts from leaves infiltrated with Zera®-VP2ep (**a**) and Zera®-VP2 (**b**) constructs at infiltration OD_600_ values of 0.5 and 1.5, respectively and harvested at 2, 3 5 and 7 days post infiltration (dpi). Expressed protein was detected with α-VP2R polyclonal antibody. Samples from negative control plants (−) were infiltrated with infiltration medium only. *Red arrows* indicate the position of the appropriately-sized expressed proteins. Lanes M represent the molecular weight marker with sizes indicated in kDa. **c** *N. benthamiana* leaves infiltrated with different *A. tumefaciens* cell concentrations. Leaves expressing Zera®-VP2ep and Zera®-VP2 were photographed at 3 and 7 dpi, respectively

Zera®-VP2ep and Zera®-VP2 were successfully cloned into pEAQ-*HT* (George Lomonossoff, JII, Norwich) to generate pEAQ-*HT*Zera®-VP2ep (Additional file 1: Figure S2a) and pEAQ-*HT*Zera®-VP2 (Additional file 1: Figure S2c) and the recombinant constructs electroporated into *Agrobacterium tumefaciens* LBA4404. Transient expression of Zera®-VP2ep and Zera®-VP2 after *Agrobacterium*-mediated infiltration of *N. benthamiana* leaves was investigated over a 7 day period, where leaf discs were harvested at 2, 3, 5 and 7 days post infiltration. Plants were infiltrated at an OD_600_ of 0.5, 1.0 and 1.5 with recombinant Zera®-VP2ep or Zera®-VP2 to examine the effects of *A. tumefaciens* cell concentration on recombinant protein expression levels. Both proteins were detected on western blots, with predicted bands of Zera®-VP2ep and Zera®-VP2 at ~16 kDa and ~120 kDa, respectively (Fig. 2a and b). Zera®-VP2ep accumulation was observed on days 2, 3 and 5, with peak accumulation observed at 3 dpi (Fig. 2a) when using an infiltration OD_600_ of 0.5. The infiltrated leaves showed chlorosis with slight necrosis at the infiltration sites at 3 dpi (Fig. 2c – top panel), after which the infiltrated leaves became more necrotic. When the cell concentration was increased to an OD_600_ of 1.0 and 1.5, the plants showed severe necrosis with blackening and drying out of the leaves (Fig. 2c – top panel). In addition the increased cell concentration resulted in no expression of Zera®-VP2ep at any of the days sampled post infiltration (results not shown).

In contrast, expression of Zera®-VP2 was only detected on western blots by using an infiltration OD_600_ of 1.5 (Fig. 2b). Expression was observed from 5 dpi onward, with the highest levels detected at 7 dpi. The high infiltration OD resulted in slight chlorosis of the leaves at 7 dpi (Fig. 2c – bottom panel), however it did not negatively affect expression of Zera®-VP2. Even though the leaves infiltrated at lower cell concentrations (Fig. 2c) displayed chlorosis, no protein was detected on western blots (results not shown).

### In situ characterisation of PBs

**Fig. 3:**
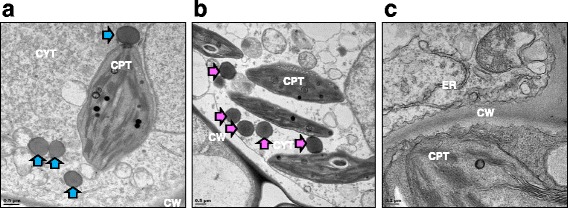
Transmission electron micrographs of protein bodies. Leaf sections were infiltrated with **a** pEAQ-*HT*Zera®-VP2ep and **b** pEAQ-*HT*Zera®-VP2 and **c** infiltration medium as a negative control. PBs for both Zera®-VP2ep (**a** – *blue arrows*) and Zera®-VP2 (**b** – *pink arrows*) were present as electron-dense structures within the cytoplasm of the infiltrated leaves. No similar structures were present in the negative control samples (**c**). *Scale bars*: **a** and **b**: 0.5 μm; **c**: 0.2 μm. CW: cell wall, CPT: chloroplast, CYT: cytoplasm, ER: endoplasmic reticulum

Having shown that optimal expression of Zera®-VP2ep and Zera®-VP2 occurred at 3 and 7 dpi respectively, based on protein band intensity on western blots, we determined whether or not these polypeptides were sequestered into PBs as designed. It has been shown that the Zera® domain containing the (PPPVHL)_8_ repeats and the Pro-X sequences allow for the accumulation of fusion proteins into membrane-bound PBs [45, 49]. TEM of embedded Zera®-VP2ep and Zera®-VP2 leaf material at 3 and 7 dpi, respectively, showed the presence of membrane-defined, spherical electron-dense PB-like structures ranging from 0.6 μm to 1 μm in size in the cytoplasm of leaves expressing Zera®-VP2ep and Zera®-VP2 (Fig. 3a and b, respectively). These electron-dense structures were embedded in the cytoplasm. No structures resembling PBs were observed in any of the negative control samples that were viewed (Fig. 3c).

### Purification of PBs

**Fig. 4:**
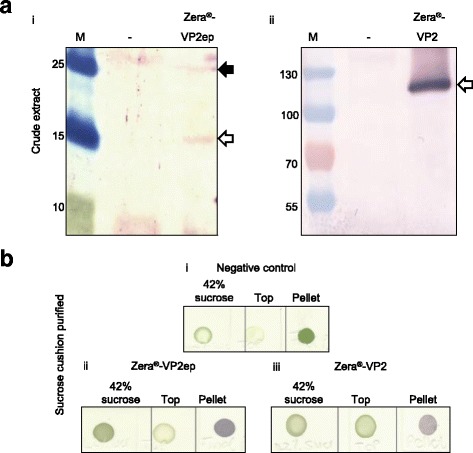
Purification of PBs. **a** Western blot analysis of crude Zera®-VP2ep (i) and Zera®-VP2 (ii) extracted protein using α-VP2R as primary antibody. *White arrows* indicate the respective proteins with the *black arrow* showing an oligomerised fusion of VP2ep. In both cases the negative control (−) was plant material infiltrated with infiltration medium only that was extracted using the same method. Lanes M indicate the molecular weight marker in kDa. **b** α-VP2R dot blots of (i) the negative control, (ii) Zera®-VP2ep and (iii) Zera®-VP2 purified using a 42% sucrose cushion

Since it was shown that Zera®-VP2ep and Zera®-VP2 successfully formed PB-like structures when expressed in *N. benthamiana*, the number of plants infiltrated was scaled up to purify PBs by ultracentrifugation through a sucrose cushion. Western blot analysis of the clarified crude protein extracts showed the presence of bands at ~16 kDa for Zera®-VP2ep (Fig. 4a [i]) and ~120 kDa for Zera®-VP2 (Fig. 4a [ii]). The detection of an additional band at ~26 kDa on the Zera®-VP2ep western blot (Fig. 4a [i], black arrow) could potentially represent dimerised fusions of Zera®-VP2ep. No bands were detected in the crude extracts from negative control plants (Fig. 4a [i] and [ii]).

Purification of the PBs using discontinuous sucrose gradients was not successful as western blot analysis of the fractions collected after ultracentrifugation showed the presence of Zera®-VP2ep and Zera®-VP2 in all the fractions, with the bulk of protein detected in the pellet (results not shown). This could be because of the variable sizes and therefore densities of the PBs, as well as the presence of soluble protein in the preparation. Accordingly, crude Zera®-VP2ep and Zera®-VP2 as well as negative control plant extracts were overlaid onto 42% sucrose cushions and centrifuged at 79 000 *× g* to facilitate collection of the dense PBs in the pellet. After centrifugation, the supernatant on top of the sucrose cushion (Top), the sucrose cushion itself (42% sucrose) and the resuspended pellets (Pellet) were analysed on dot blots probed with α-VP2R serum (Fig. 4b). Fractions collected from the negative control showed no colour development in either the pellet, the 42% sucrose fraction or the top fraction (Fig. 4b [i]). Dot blots of Zera®-VP2ep and Zera®-VP2 showed a positive reaction with the pellet sample, indicating successful pelleting of the recombinant PBs through the sucrose cushion (Fig. 4b [ii] and [iii], respectively); no protein was detected in either the top or 42% sucrose fractions.

The purified PBs were found to be very stable as well as insoluble. Dot blot analysis of pelleted Zera®-VP2ep and Zera®-VP2 always showed the presence of oligomerised proteins in the denatured samples, even after treating the purified proteins at 90 °C with 500 mM DTT in order to denature them (results not shown). It was further investigated whether the sequential extraction process described by Joseph et al. [43] would result in the solubilisation of the PBs. The first solubilisation step facilitates the extraction of soluble and membrane-bound proteins, after which the second step extracts proteins that are linked by disulphide bridges. The final heating step should theoretically result in solubilisation of the PB cores [43]. After treatment with SDS and DTT, the PBs were always present in the final insoluble pellet (data not shown). Since the PBs were intended for immunogenic studies in mice, it was not desirable for the extraction buffers to contain reagents such as DTT or SDS, as these might be detrimental to the health of the animals. Therefore, the pellet obtained after ultracentrifugation was washed with buffer PBP3 containing 10% sucrose to stabilise the PBs and protein concentrations of Zera®-VP2ep and Zera®-VP2 PBs were calculated to be ~ 69.5 mg and ~ 35 mg total protein per kilogram of fresh leaf material, respectively.

### Humoral immune response of PB vaccine candidates

**Fig. 5:**
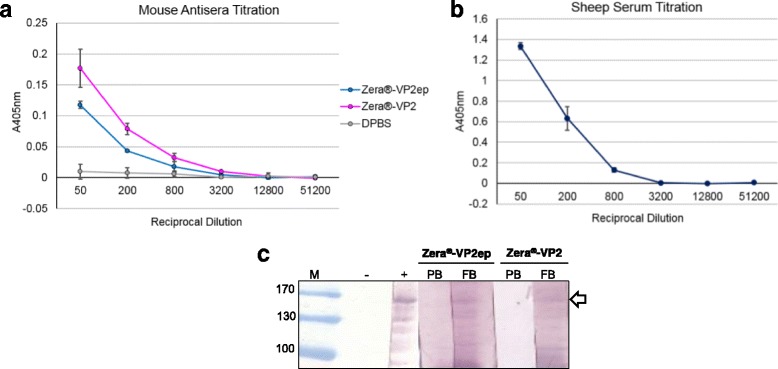
Analysis of serum from immunised mice. **a** Titration of the mouse antisera produced Zera®-VP2ep (*blue line*), Zera®-VP2 (*pink line*) and DPBS (negative control, *grey line*) vaccine candidates as well as titration of positive control sheep serum produced against BTV-8 VLPs [20] to validate the indirect ELISA (**b**). The *markers* indicate the mean value of triplicate samples from both animal experiments, and *error bars* indicate the standard deviation. (c) Western blot detection of the *E. coli*-expressed VP2 fusion protein with 1:100 dilution of pooled mice sera from animals vaccinated with the Zera®-VP2ep and Zera®-VP2 vaccines. Lane M represents the molecular weight marker. The negative control (−) was performed with no primary antibody and sheep serum obtained from BTV-8 VLP vaccinated sheep [20] was used as the positive control (+). Lanes PB and FB represent the pre-and final bleed sera respectively. The *white arrow indicates* the *E. coli*-expressed VP2 fusion protein at ~163 kDa. PB – prebleed; FB – final bleed

In order to determine whether the purified PBs stimulate antibodies which are VP2-specific, mice were inoculated with the Zera®-VP2ep and Zera®-VP2 PB candidate vaccines at 10 μg per dose and the humoral anti-VP2 immune responses analysed by indirect ELISA, using *E. coli*-produced BTV-8 VP2 fused to a trigger factor chaperone as coating antigen. Initial analysis of the pre-and final bleed sera of the individual mice showed the presence of anti-VP2 antibodies in only the final bleed sera of mice vaccinated with both the Zera®-VP2ep and Zera®-VP2 candidate vaccines, therefore mouse sera from each group were pooled (5 mice/vaccine) for analysis of the anti-VP2 binding titres. The antibody binding titres (Fig. 5a) are expressed as the reciprocal of the maximum serum dilution containing absorbance values that were three times greater than the corresponding pre-bleed serum at 1:50. No anti-VP2 response was detected for the vaccine pre-bleeds (results not shown) and the negative control vaccine (DPBS, Fig. 5a). The positive control indirect ELISA using sheep serum (from sheep vaccinated with plant-produced BTV-8 VLPs [20]) showed an anti-VP2 response (Fig. 5b), thereby validating the indirect ELISA used to determine anti-VP2 titres of the Zera® vaccine candidates tested in this study.

The Zera®-VP2ep vaccine elicited anti-VP2 titres of 800, with an OD_405_ of ~0.177 (Fig. 5a, blue line) compared to an OD_405_ of ~0.0039 obtained for the corresponding pre-bleed serum. In addition, the Zera®-VP2 vaccine candidate elicited anti-VP2 binding titres of 3200 at an OD_405_ of ~0.0097 (Fig. 5a, pink line), compared to an OD_405_ value of ~0.0016 obtained for the corresponding pre-bleed serum. Statistical analysis of the anti-VP2 responses elicited by the Zera®-VP2ep and Zera®-VP2 vaccine candidates, showed that the antibody levels were significantly higher for both vaccines compared to the DPBS negative control (KW; H = 22.002, N = 30, *p* < 0.0001).

The ability of the antibodies produced against Zera®-VP2ep and Zera®-VP2 to recognise the BTV-8 VP2 fusion protein was further analysed by evaluating mouse serum from mice inoculated with Zera®-VP2ep and Zera®-VP2 vaccine candidates on western blots using the *E. coli*-produced BTV-8 VP2 fusion protein as antigen. Individual pre-and final bleed sera from both experiments were pooled for each vaccine candidate and analysed for anti-VP2 responses (Fig. 5c).

The *E. coli*-expressed VP2 fusion protein (~163 kDa, white arrow) was detected with the positive control sheep serum raised against plant-produced BTV-8 VLPs and the final bleed serum obtained from Zera®-VP2ep- and Zera®-VP2-vaccinated mice (Fig. 5c). Non-specific protein bands smaller than the ~163 kDa VP2 band were detected with all the serum samples, including the positive control. These bands possibly represent truncated versions of the *E. coli*-expressed VP2. It has been shown that heterologous expression in *E. coli* often results in the expression of truncated versions of the expressed proteins due to abundant codons found in heterologous genes which cause translational stalling and termination of protein expression [50]. No non-specific protein bands were detected with the pre-bleed sera.

## Discussion

The incursion of BTV-8 into northern Europe in 2006 resulted in significant economic losses due to the mortality of affected livestock, but more importantly due to the ban of ruminant trade between affected and non-affected regions [51]. In an effort to limit the direct losses incurred with BTV infection and to minimize circulation of the virus, vaccination of livestock using four monovalent modified live virus vaccines obtained from South Africa was undertaken by European countries [9, 11]. Many vaccine candidates have been developed against BT – with varying degrees of success [19]. Recombinant vaccines are promising vaccine candidates as they have the potential to be safer than attenuated vaccines with no risk of reassortment, and they can be DIVA compliant [52, 53].

Immunologically, VP2 is the most important BTV protein [54–58], and inocula consisting of VP2 on its own or combined with VP5 have been tested as possible vaccine candidates, with variable results [11, 59]. Even though VP2 subunit vaccines provided protection at lower doses, no neutralising antibodies were detected after vaccination. It is therefore desirable to increase immunogenicity of these vaccine candidates; this could potentially be achieved by fusion of VP2 to Zera® which results in formation of particulate proteins which are partly protected from extracellular enzymatic degradation. These particulate proteins indirectly or directly target antigen presenting cells (APCs) by either accessing the specific endosomal uptake function of APCs or by interaction with specific dendritic cells, thereby enhancing the immune response [60]. Moreover, it has been postulated that the N-terminus of Zera® potentially possesses intrinsic adjuvant activity, thereby further enhancing the immune response [35, 36, 47, 61].

In an effort to find alternative, possibly multivalent vaccine candidates for BTV, we undertook to design and express two novel second-generation BTV vaccines in *N. benthamiana* by means of *Agrobacterium*-mediated transient recombinant protein expression. The first vaccine is based on synthetic B-cell epitopes predicted for multiple serotypes of VP2 by using online computational methods; the second vaccine is based on incorporation of the full length BTV-8 VP2 only. By utilising the immunogenic properties of only VP2, these vaccine candidates were aimed at being DIVA-compliant using current commercially available serological diagnostic tests which are dependent on detecting the presence of the group-specific antigen VP7 [2, 22].

The putative multi-epitope (VP2ep) and full length BTV-8 VP2 were both fused to Zera® to induce accumulation of the proteins in dense PBs called StorPro® organelles (ZIP Solutions, Spain). Both Zera®-VP2ep and Zera®-VP2 proteins were successfully expressed in plants, and it was shown using TEM that both these constructs directed the accumulation of BTV-specific proteins in electron-dense granules that were surrounded by a membrane, within the cytoplasm of plant cells. These structures were similar in size and morphology to epidermal growth factor PBs produced in SF9 insect cells [45]. Two steps have been described for the formation of PBs: (i) early synthesized Zera® fusion polypeptide chains promote self-assembly of Zera®, resulting in the origin of PBs and thereafter (ii) the continued synthesis of the fusion protein at later stages results in the PB growing by incorporating Zera® fusions into the PBs that were formed during the early stages of expression [42]. The different sizes that were observed for the Zera®-VP2ep and Zera®-VP2 PBs could be attributed to the occurrence of the two-step process described above.

We attempted to purify Zera®-VP2ep and Zera®-VP2 PBs on discontinuous sucrose gradients. Analysis of the fractions collected after ultracentrifugation showed that the proteins of interest were present in all the fractions, including the pellet. These results could be attributed to the different sizes and therefore densities of early and mature protein bodies. Similar results were obtained by Geli et al. [49] after subcellular fractionation of transgenic plants expressing γ-zein. Therefore, a 42% sucrose cushion was used instead to pellet the PBs in the plant extract, this made the PBs very easy to partially purify. The pelleted PBs were extremely stable and insoluble, even after treatment with SDS, DTT and heat. The sequestration of proteins in PBs that are membrane-delimited has been shown to protect fusion proteins from proteolytic degradation [45]. The stability of the PBs is ideal for vaccines, as it means the vaccine candidates can be stored at 4 °C for extended periods of time without the risk of degradation or proteolysis.

Both the Zera®-VP2ep and Zera®-VP2 fusion proteins were successfully detected with rabbit serum raised against the full-length VP2 fusion protein. These results showed that antibodies in the sera were able to recognise and bind to the synthetic epitope, suggesting that regions encoding immunogenic epitopes were accurately selected for design of this vaccine candidate. These results are promising, as success of the epitope-based vaccine could obviate the need for vaccination with a mixture of multiple serotypes since it contains putative epitopes for at least eight BTV serotypes.

Preliminary tests to analyse the immunogenicity of the Zera®-VP2ep and Zera®-VP2 candidate vaccines were carried out in mice. This proof of concept study showed that both the Zera®-VP2ep and Zera®-VP2 candidate vaccines elicited significant anti-VP2 humoral immune responses when compared to the negative control. Moreover, the reaction of the antibodies in both ELISA and western blots means the antibodies elicited in vaccinated mice would bind native protein as well as denatured protein, meaning they have the potential to protect against infection. Future experiments, such as neutralization assays and challenge experiments of immunized animals, have to be carried out in order to further characterize the immune response obtained with these vaccine candidates.

## Conclusions

In summary, Zera®-VP2ep and Zera®-VP2, two novel protein body vaccine candidates encoding the predicted putative B-cell epitopes of VP2 and full-length codon-optimised VP2 fused to Zera® to make particulate vaccines were successful in eliciting anti-VP2 humoral immune responses in mice. These novel PB vaccine candidates have shown great potential in this proof of concept study for the use of BTV vaccines, as they are quick and simple to produce, extremely stable and DIVA compliant, making them ideal as rapid response vaccines.

## Methods

### *In silico* epitope prediction of VP2 and construction of the fusion product Zera®-VP2ep

Eight full-length BTV VP2 amino acid sequences (BTV-1: ADI79209; BTV-2: CA079950; BTV-6: ADI49552; BTV-8: AM498052; BTV-10: JN704634; BTV-13: AAX48783; BTV-17: AAB30550 and BTV-23: AAA56867) for proof of concept were selected from GenBank and the most likely epitopes of each of these sequences were predicted with COBEpro [[27], http://scratch.proteomics.ics.uci.edu/], an online program for the prediction of continuous B-cell epitopes. The VP2 amino acid sequences were aligned using ClustalW2 (https://www.ebi.ac.uk/Tools/msa/clustalw2/) and two predicted B-cell epitope regions whose amino acid compositions corresponded to most of the aligned VP2 sequences were selected for inclusion into the synthetic epitope-based vaccine. A third homologous region was also included in the synthetic epitope-based vaccine sequence.

The plasmid pZera®1 [45] obtained from ZIP Solutions was used as a template for the PCR amplification of *Zera®*. Primers were designed to introduce 5′ *Age*I and 3′ *Nco*I restriction enzyme sites to the gene termini (Table 1; Zera®-FP and Zera®-RP).

**Table 1:** Primers and oligomers used for PCR of individual genes and the assembly PCR of fusion products

Gene/Oligomer	Size (bp)	Primer	Restriction sites (5′ / 3′)	5′ – 3′ sequence^a^	Tm (°C)
Zera®	352	Zera®-FP^b,c^ Zera®-RP	*Age*I *Nco*I	GC**ACCGGT**ATGAGGGTGTTGCTCGTT	62.7 58.3
				GC**CCATGG**CTGGCACGGGCTTGGAT	
VP2ep	70	VP2ep-F VP2ep-R	*Mlu*I *Xma*I	GC**ACGCGT**TTCCCAGAGCACCTTCGACACGATGTAACAAAT	76.7 79.8
				GC**CCCGGG**TCCACGCAGGCAGGGATACGGATTTGTTACATC	
Linker			*Nco*I/*Mlu*I	**CCATGG**GAAGCGGCGGCGAAA**ACGCGT**	75.9
		Linker-VP2ep-FP^b^		GAAGCGGCGGCGAAAACGCGTTTCCCAGAGCACCTT	77.3
		Linker-Zera®-RP^b^		TTTCGCCGCCGCTTCCCATGGTCTGGCACGGGCTTG	81.4
		VP2ep-RP^b,c^	*Xma*I	GC**CCCGGG**TCCACGCAGGCAGGG	56.8
*VP2*	~2800	cTPVP2coF	*Mlu*I	GTGG**ACGCGT**TAGGTGC**ATG**GAAGAACTCGCTATCCCAA	56
		cVP2coR	*Xho*I	GC**CTCGAG**TCAAACGTTGAGGAGCTTAGTAAG	54
Sequencing primers		pEAQ-FP pEAQ-RP		GACGAACTTGGAGAAAGATTGTTAAGC	61.2 62.3
				AACCAGAGTTAAAGGCCTCGAGC	

^a^The restriction enzyme sites are underlined and in bold

^b,c^Primers used for first and second stage assembly PCR of Zera®-VP2ep

The putative multi-epitope sequence - *VP2ep* - was designed to contain 5′ *Mlu*I and 3′ *Xma*I restriction enzyme sites. Two oligomers - VP2ep-F and VP2ep-R (Table 1) - consisting of 41 bp each were designed and synthesized with 12 complementary base pairs to facilitate fusion of the fragments during assembly PCR to yield *VP2ep*. The assembly PCR reaction consisted of 1 μM each of VP2ep-F and VP2ep-R, 200 μM dNTPs, 1× Buffer A (Kapa Biosystems) and 1 U KAPA Taq DNA polymerase (Kapa Biosystems). The two oligomers were assembled by initial denaturation of the DNA at 94 °C for 5 min, followed by 20 cycles of denaturing at 93 °C for 1 min and annealing and elongation at 65 °C for 30 s. A final elongation step was carried out for 5 min at 72 °C.

*Zera®* was fused to *VP2ep* with a short universal peptide linker - EAAAK (Table 1). Linker primers containing regions that were complementary to both the linker, *Zera®* and *VP2ep* were designed (Table 1; Linker-VP2ep-FP and Linker-Zera®-RP) to facilitate assembly of the fusion product.

Assembly PCR of *Zera®-VP2ep* was carried out in two stages. Additional file 2: Figure S1 illustrates the fusion product *Zera®-VP2ep*, including the primers that were used in first and second stage assembly PCR reactions. The first stage PCR reaction consisted of 50 ng of each of the following DNA templates: *Zera®*, *VP2ep* and the linker. The remainder of the reaction contained 1 μM of each primer (Zera®-FP, Linker-VP2ep-FP, Linker-Zera®-RP, VP2ep-RP), 200 μM dNTPs, 1× Buffer A and 1 U KAPA Taq DNA Polymerase. PCR was carried out with an initial denaturing step at 94 °C for 2 min, followed by 25 cycles of denaturing at 93 °C for 1 min and annealing and elongation at 72 °C for 30 s. A final elongation step was carried out for 5 min at 72 °C. The assembled PCR product was used as a template in the second stage PCR. The second stage PCR reaction contained 50 ng of the template DNA, 1 μM each of Zera®-FP and VP2ep-RP, 200 μM dNTPs, 1× Buffer A and 1 U KAPA Taq DNA Polymerase. Second stage cycling conditions were similar to the first stage assembly PCR conditions, however only 20 cycles of amplification were carried out and the annealing temperature was decreased to 65 °C.

### Cloning into the plant-expression vector pEAQ-*HT*

*Zera®-VP2ep* was directionally cloned into the pEAQ-*HT* [62] plant expression vector using *Age*I and *Xma*I restriction enzyme sites to yield pEAQ-*HT*Zera®-VP2ep (Additional file 1: Figure S2a). DH5-α chemically competent *E. coli* cells (E. cloni™, Lucigen) cells were transformed [63] with the plasmid construct and recombinant clones were selected using kanamycin resistance (50 μg/mL). Recombinant clones were screened by colony PCR using Zera-FP and VP2ep-RP primers. Recombinant clones were verified by sequencing with pEAQ-*HT* primers (Table 1).

The full length BTV-8 *VP2* gene sequence was codon-optimised for expression in *N. benthamiana* in a previous study [64]. BTV-8 *VP2* was excised from the vector backbone using restriction enzyme digestion with *Mlu*I and *Xho*I. Likewise, VP2ep was excised from the plasmid pEAQ-*HT*Zera®-VP2ep (Additional file 1: Figure S2b) so that it could be replaced with BTV-8 *VP2* to create pEAQ-*HT*Zera®-VP2 (Additional file 1: Figure S2c). Plasmids were transformed into *E. coli* as described above and recombinant clones were screened by colony PCR using *VP2* gene-specific primers (Table 1). The recombinant plasmid was verified by sequencing with the pEAQ-*HT* primers (Table 1).

### *A. tumefaciens*-mediated transient expression of Zera®-VP2ep and Zera®-VP2

pEAQ-*HT*Zera®-VP2ep and pEAQ-*HT*Zera®-VP2 were transformed into electrocompetent *Agrobacterium tumefaciens* LBA4404 using the method described by Maclean et al. [65]. Transformants were selected on Luria Bertani (LB) media plates containing 30 μg/mL kanamycin and 50 μg/mL rifampicin. Successful transformation was confirmed with colony PCR.

Starter cultures of *A. tumefaciens* harbouring the pEAQ-*HT*Zera®-VP2ep and pEAQ-*HT*Zera®-VP2 plasmids were supplemented with 30 μg/mL kanamycin, 50 μg/mL rifampicin and 2 mM MgSO_4_ and grown in LB media as described by Maclean et al. [65]. The starter cultures were used to inoculate induction medium supplemented with 30 μg/mL kanamycin, 50 μg/mL rifampicin, 20 μM acetosyringone and 2 mM MgSO_4_. The cultures were propagated O/N at 27 °C with agitation, after which the cells were prepared for syringe infiltration into six-week-old *N. benthamiana* leaves. Time-trials were carried out to evaluate at what cell density and which day the best expression of Zera®-VP2ep and Zera®-VP2 occurred, after which expression was scaled up.

### In situ TEM of Zera®-VP2ep and Zera®-VP2 PBs

Recombinant pEAQ-*HT*Zera®-VP2ep or pEAQ-*HT*Zera®-VP2 plasmids were syringe-infiltrated into six-week-old *N. benthamiana* plants and protein was expressed for 3 and 7 days, respectively.

Embedding and sectioning of the leaf material was carried out according to the method described by van Zyl et al. [64]. Grids were viewed using a Technai G2 transmission electron microscope (FEI).

### Screening for Zera®-VP2ep and Zera®-VP2 expression in *N. benthamiana*

For small-scale expression three leaf discs were harvested at 2, 3, 5 and 7 dpi and ground up in liquid nitrogen. The leaf material was resuspended in 70 μL per disc of buffer PBP3 (100 mM Tris [pH8], 50 mM KCl, 6 mM MgCl2, 10 mM EDTA, 0.4 M NaCl and 1× Complete Mini EDTA-free protease inhibitor cocktail [Roche]). The leaf extracts were clarified for 5 min at 15 000 *× g* on a bench top centrifuge.

After large scale expression of Zera®-VP2ep and Zera®-VP2, leaves were harvested at 3 and 7 dpi, respectively and was homogenised with a Waring-type blender in five volumes ice cold PBP3 buffer containing 10% sucrose and 1 × Complete Mini EDTA-free protease inhibitor cocktail. The crude plant extract was incubated for 1 h at 4 °C with gentle agitation, after which it was filtered through Miracloth™ (Merck) and further clarified by centrifugation at 10 000 *× g* for 10 min at 4 °C.

### Purification of protein bodies (PBs)

The clarified crude plant extract was overlaid onto 5 mL of a 42% sucrose cushion prepared in buffer PBP3 and ultracentrifuged for 2 h at 79 000 *× g* (Beckman SW32Ti rotor) at 4 °C. The pellet containing PBs was resuspended in 300 μL buffer PBP3 containing 10% sucrose. Purified PBs were stored at 4 °C. Total protein yield was quantified using the *DC* Protein Assay (Bio-Rad) according to the manufacturer’s instructions.

### *E. coli*-based expression of BTV-8 VP2 and antibody production in rabbits

Since there is no commercially available antibody for BTV VP2, the BTV-8 VP2 protein (~163 kDa) was expressed as a fusion product with a trigger factor chaperone and a translation enhancing element to achieve high level, soluble expression in *E. coli* (http://www.takara-bio.com). The BTV-8 VP2 fusion protein was expressed for 24 h using the pCold™TF Cold Shock Expression System (TAKARA) according to the manufacturer’s instructions. Inclusion bodies were purified from the *E. coli* cell pellet using Bugbuster® (Novagen, USA) according to the manufacturer’s instructions. For antibody production in rabbits, the resuspended VP2 fusion protein inclusion bodies were dialysed O/N at 4 °C using dialysis tubing with a molecular weight cut-off of 10 kDa (Thermo Fischer Scientific, USA) in 2 L sterile Dulbecco’s Endo-free PBS (Sigma-Aldrich, USA). The VP2 fusion protein was quantified with gel densitometry using a bovine serum albumin (BSA, Sigma-Aldrich) protein standard.

Deltamune (Pty) Ltd. (South Africa) carried out inoculation and production of the polyclonal antibody in rabbits, protocols were approved by the Deltamune Animal Ethics Committee prior to being carried out. For the primary inoculation rabbits were injected subcutaneously with 32 μg of the BTV-8 VP2 fusion protein in the presence of Incomplete Freund’s Adjuvant (IFA, Difco, #263910). This was followed by three subsequent booster vaccinations (in the presence of IFA); the first booster was administered two weeks after the initial injection and thereafter the second and third boosters followed one week after the other.

Rabbit serum (α-VP2R) obtained from Deltamune was used as primary antibody for western– and dot blot analysis of Zera®-VP2ep and Zera®-VP2.

### Western and dot blot analysis of Zera®-VP2ep and Zera®-VP2

For western blot analysis, the plant extracts were incubated at 90 °C for 10 min in 5 × DTT sample application buffer (250 mM Tris-Cl [pH6.8], 500 mM DTT, 10% sodium dodecyl sulphate [SDS], 0.3 mM bromophenol blue and 10% glycerol). Zera®-VP2ep and Zera®-VP2 proteins were separated on 15 and 8% SDS polyacrylamide gels, respectively, and transferred onto nitrocellulose by semi-dry electroblotting. Dot blots were carried out to detect purified PBs. A volume of 5 μL of the purified protein was dropped onto nitrocellulose membranes and dried completely, after which dotblots were treated the same as western blots.

Zera®-VP2ep and Zera®-VP2 was detected with using a 1: 2000 dilution rabbit-raised anti-VP2 (BTV-8) polyclonal antibody (α-VP2R). These antibodies were detected with 1:5000 alkaline phosphatase-conjugated anti-rabbit antibody (Sigma-Aldrich). Detection was performed using BCIP/NBT (KPL) substrate.

### Immunization of mice

Approval for this study was granted by the Animal Research Ethics Committee at the University of Cape Town (AEC# 011–016). Female BALB/c mice (6–8 weeks old, obtained from South African Vaccine Producers) were subcutaneously immunised with the partially purified Zera®-VP2ep and Zera®-VP2 candidate vaccines at 10 μg per dose (5 mice per vaccine). Dulbecco’s Phosphate Buffered Saline (DPBS, Sigma) was used as a negative control. Pre-bleeds were collected prior to vaccination (day 0) and mice were boosted on Day 28 with doses containing 10 μg of the appropriate antigens. Final bleeds were obtained via cardiac puncture at Day 56. The immunization study was repeated twice.

### Indirect ELISA for detection of anti-VP2 antibodies

The BTV-8 VP2 fusion protein was expressed and extracted according to the method described above for antibody production. The fusion protein was used as coating antigen in indirect ELISAs to test mouse serum and serum obtained from sheep vaccinated with BTV-8 VLPs [20] was used as positive control primary antibody to validate the ELISA.

The anti-VP2 response of the candidate vaccines was determined by indirect ELISA. Ninety-six-well Maxisorp® microtitre plates (Nunc) were coated with 100 μL/well (1 μg) of the *E. coli*-produced BTV-8 VP2 fusion protein diluted in coating buffer (10 mM Tris, pH 8.5) and incubated O/N at 4 °C. The plates were blocked with TBS blocking buffer (5% non-fat dry milk in 1 × TBS [50 mM Tris, 150 mM NaCl, pH 7.5]) for 2 h at room temperature after which it was washed with 1 × TST buffer (1 × TBS (pH 7.5), 0.05% Tween®20). To evaluate the anti-VP2 immune response elicited by each mouse, the pre- and final bleed sera were diluted 1:50 in 100 μL TBS blocking buffer and incubated for 2 h at room temperature. Plates were washed with 1 × TST buffer and 100 μL goat anti-mouse IgG alkaline phosphatase conjugate (1:10 000, Sigma) diluted in blocking buffer was added per well and incubated for 1 h at 37 °C. After incubation the plates were washed with 1 × TBS (pH 9) buffer and 200 μL SIGMAFAST™ p-Nitrophenyl phosphate (pNPP, Sigma) to detect the secondary antibody. The plates were developed in the dark for 30 min after which the absorbance was read at 405 nm.

To determine the anti-VP2 binding titres, mouse sera from vaccine candidates were pooled into vaccine groups (5 mice/vaccine) for analysis. Final bleed mouse sera were diluted in TBS blocking buffer in a 4-fold series in triplicate ranging from a 1:50 dilution to 1:51 200. Mouse sera from the mice vaccinated with DPBS served as a negative control. Positive control wells to validate the ELISA contained sheep serum [20] and blank wells with no antibody were included for background control in all the ELISAs. The indirect ELISA was carried out as described above and the anti-VP2 binding titres were expressed as a reciprocal of the maximum serum dilution which produced absorbance values that were three times greater than the corresponding pre-bleed serum diluted at 1:50. To calculate the statistical significance of the final bleed anti-VP2 responses between the vaccines and the negative control, a Kruskal-Wallis (KW) test was performed (due to non-normal and heteroscedastic data) to compare medians among groups. Statistical analysis was carried out using STATISTICA 64 (StatSoft, Inc.).

### Western blot detection with Zera®-VP2ep and Zera®-VP2 mouse serum

The *E. coli*-produced BTV-8 VP2 antigen was separated on 8% SDS polyacrylamide gels and western blots were carried out as described above. Individual pre- and final bleed sera from both repeat immunization experiments were pooled into vaccine groups (5 mice per vaccine) and diluted 1:100 in blocking buffer for detection of *E. coli*-produced VP2. 1:100 BTV-8 VLP sheep serum was used as positive control [20]. Sera was detected with either anti-mouse IgG or anti-goat/sheep alkaline phosphatase-conjugated secondary antibodies, respectively.

## Additional files

Additional file 1: Figure S2. Schematic representation of the Zera®-fused constructs. (a) pEAQ-*HT*Zera®-VP2ep, and (c) pEAQ-*HT*Zera®-VP2. The pEAQ-*HT*Zera® (*Mlu*I / *Xho*I) backbone is shown in (b). Zera® is represented by the pink box, the linker sequence by the blue box and VP2ep and VP2 are represented by the green and orange boxes, respectively. (PPTX 38 kb)

Additional file 2: Figure S1. Schematic representation of the primers used to create the fusion product Zera®-VP2ep by assembly PCR. (PPTX 33 kb)
