# Supplementary material for: Development of plant-produced protein body vaccine candidates for bluetongue virus
Source: BMC Biotechnol. 2017 May 30;17:47. doi: 10.1186/s12896-017-0370-5 (PMC5450216; doi:10.1186/s12896-017-0370-5)
Supplement: Supplementary file 1 — Schematic representation of the Zera®-fused constructs. (a) pEAQ-HTZera®-VP2ep, and (c) pEAQ-HTZera®-VP2. The pEAQ-HTZera® (MluI / XhoI) backbone is shown in (b). Zera® is represented by the pink box, the linker sequence by the blue box and VP2ep and VP2 are represented by the green and orange boxes, respectively. (PPTX 38 kb) [file 12896_2017_370_MOESM1_ESM.pptx]

## Slide 1
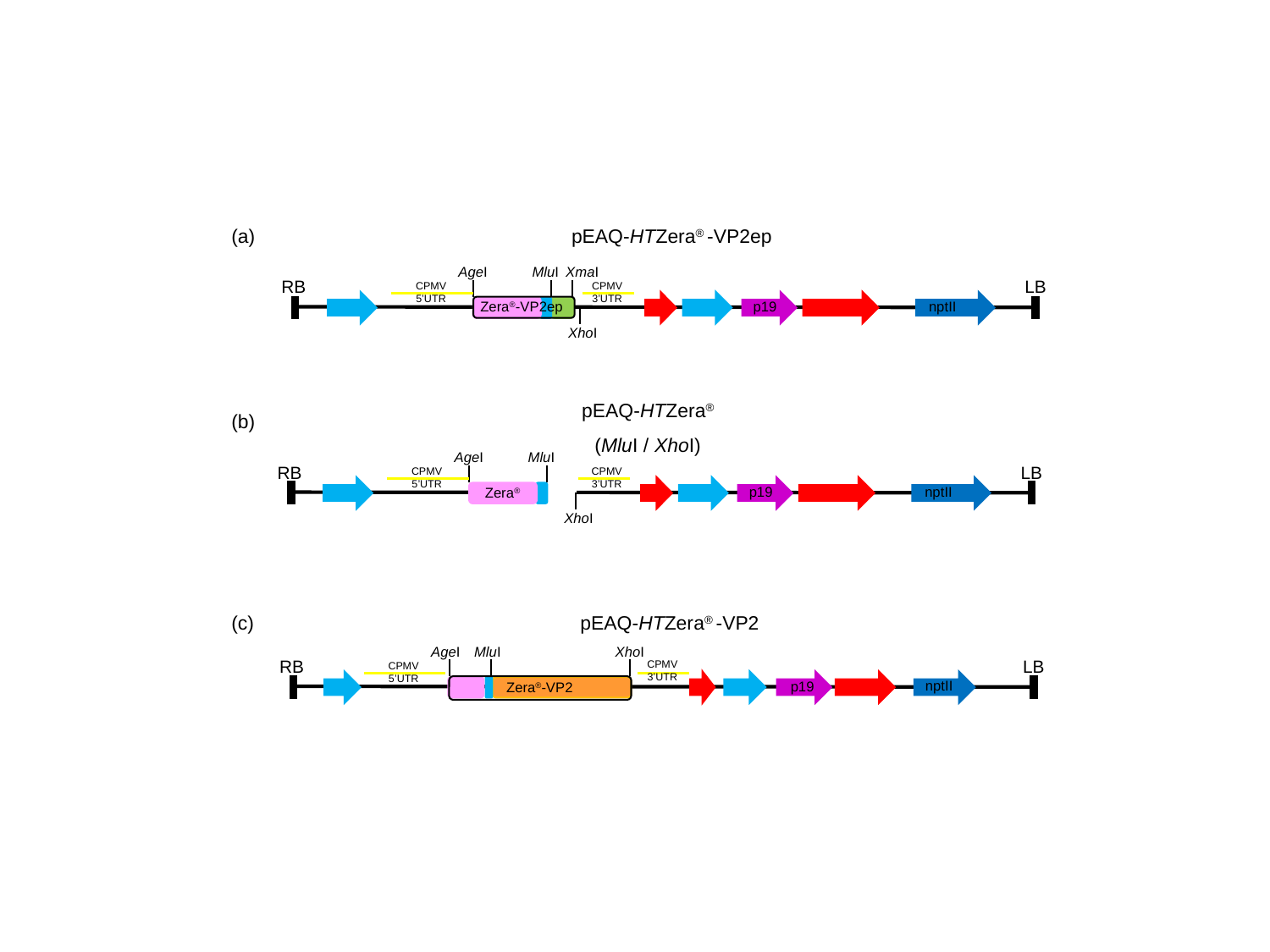

(a)
pEAQ-HTZera® -VP2ep
AgeI
MluI
XmaI
RB
LB
Zera®-VP2ep
p19
nptII
XhoI
CPMV 5’UTR
CPMV 3’UTR
pEAQ-HTZera® (MluI / XhoI)
AgeI
MluI
RB
LB
p19
nptII
XhoI
CPMV 5’UTR
CPMV 3’UTR
pEAQ-HTZera® -VP2
AgeI
MluI
XhoI
RB
LB
nptII
p19
Zera®-VP2
CPMV 3’UTR
CPMV 5’UTR
(b)
(c)
Zera®
