# Supplementary material for: Development of plant-produced protein body vaccine candidates for bluetongue virus
Source: BMC Biotechnol. 2017 May 30;17:47. doi: 10.1186/s12896-017-0370-5 (PMC5450216; doi:10.1186/s12896-017-0370-5)
Supplement: Supplementary file 2 — Schematic representation of the primers used to create the fusion product Zera®-VP2ep by assembly PCR. (PPTX 33 kb) [file 12896_2017_370_MOESM2_ESM.pptx]

## Slide 1
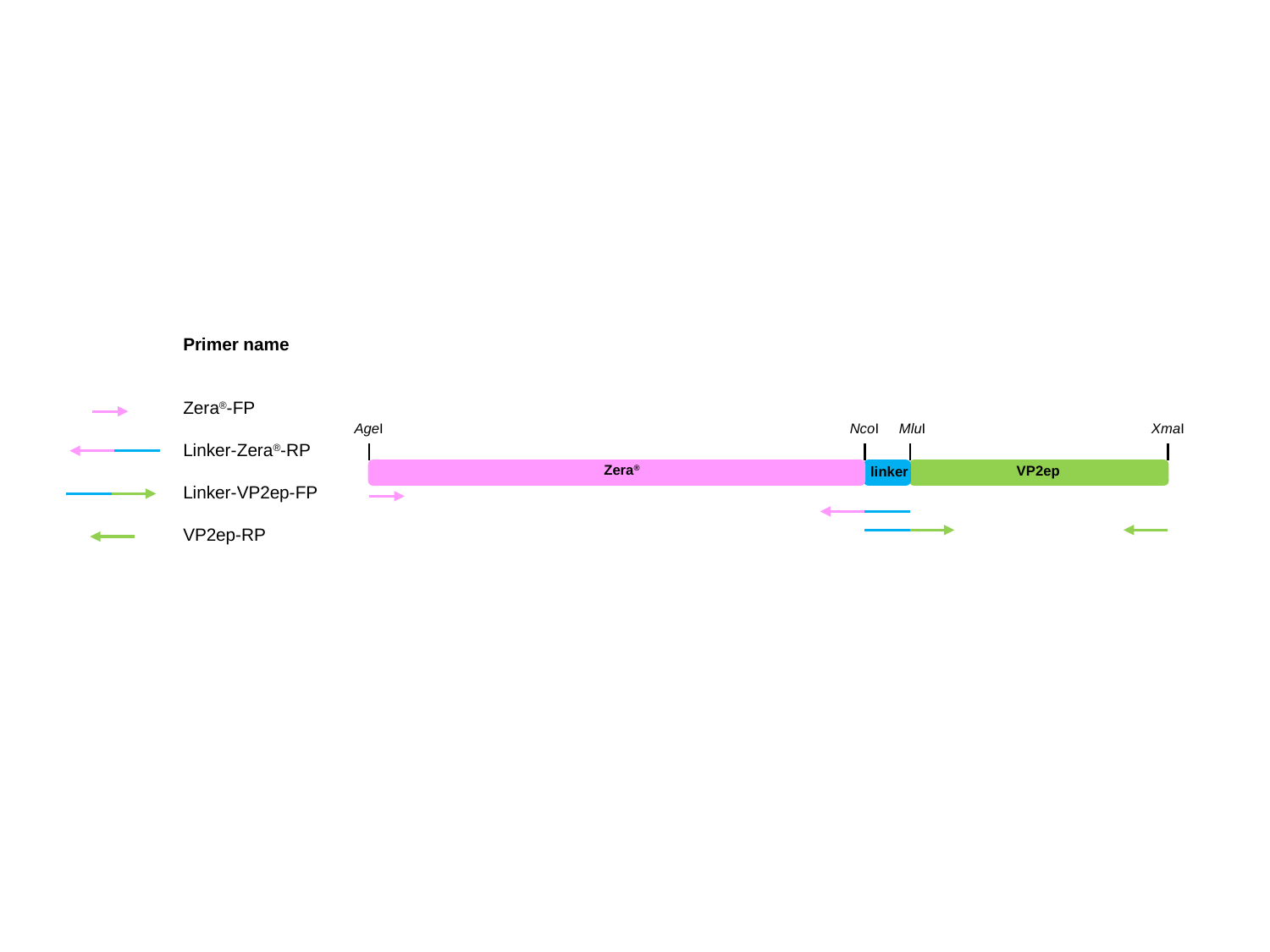

Primer name
Zera®-FP
Linker-Zera®-RP
Linker-VP2ep-FP
VP2ep-RP
AgeI
NcoI
MluI
XmaI
Zera®
VP2ep
linker
